# The human genome harbours widespread exclusive yin yang haplotypes

**DOI:** 10.1038/s41431-023-01399-5

**Published:** 2023-06-12

**Authors:** David Curtis, William Amos

**Affiliations:** 1 UCL Genetics Institute, UCL, Darwin Building, Gower Street, London, WC1E 6BT UK; 2 Department of Zoology, Downing Street, Cambridge, CB2 3EJ UK

**Keywords:** Genetic variation, Population genetics

## Abstract

There have been reports of examples of exclusive yin yang haplotypes, differing at every locus, but there has been no systematic search for them. Unphased whole genome sequence data for 2504 unrelated 1000 Genomes subjects was searched for chains of SNPs having global minor allele frequency (MAF) > =0.1 made up of at least 20 SNPs in complete linkage disequilibrium with each other and with no pair being separated by more than 9 other SNPs. The global distribution of these haplotypes was investigated, along with their ancestral origins and associations with genes and phenotypes. A number of previously unrecognised repeats were noted, flagged by all or most subjects being called as heterozygotes, and these were discarded. There were 5114 exclusive yin yang haplotypes each consisting of on average 34.8 SNPs, each spanning on average 15.7 kb and cumulatively covering 80 Mb. Although for some haplotypes the MAF varied markedly between populations the average global fixation index was similar to that for SNPs elsewhere in the genome and there was no evidence of enrichment for genes or gene ontologies. For all but 92 haplotypes there were partial forms present in the chimpanzee and/or Neanderthal genome, indicating that they had been formed in a gradual process but that intermediate haplotypes were now absent from modern humans. Exclusive yin yang haplotypes cover over 2% of the human genome. The mechanisms accounting for their formation and preservation are unclear. They may serve as useful markers of the dispersal of chromosomal regions through human history.

## Introduction

A yin yang haplotype pair consists of two haplotypes which differ at every constituent locus [1]. As we have discussed previously, such haplotypes are not expected to become common as a consequence of a process of serial single mutations, in which each new variant occurs on the background of a single pre-existing haplotype [2]. We and others have previously reported that such haplotypes, variously defined, are perhaps surprisingly frequent [1–3]. We have also reported a case of a 5.9 kb haplotype consisting of 8 rare missense variants within the *ABCA13* gene which has a worldwide distribution such that all 1000 Genome subjects carry either all alternate alleles or none [4]. A much longer pair of yin yang haplotypes, which accounted for a large proportion of all observed haplotypes, consisted of 284 variants stretching over 1 Mb and encompassed *GPHN*, the gene for gephyrin, though did not include coding variants within it [5]. The authors reported that both haplotypes in the pair differed from the ancestral haplotype and noted that it was unclear how such a genetic anomaly arose, though speculated that it could have occurred as a result of selection pressures on two independent mutations, each of which might have influenced gene expression.

By contrast, Dutta and colleagues proposed that the high frequency of non-exclusive yin yang pairs observed among common haplotypes might reflect a “Great Admixture” event between two ancestral populations [3]. They proposed a model in which two distinct ancestral populations existed over hundreds of thousands of years, with the initial separation between them being about 1.5 times older than the separation of archaic humans from Neanderthals. Subsequently admixture between the two populations occurred to give rise to the common variants and haplotype structures we observe today and they estimate the time of the “Great Admixture” to be roughly 300–100 thousand years ago. Such a model would not only account for the preponderance of yin yang haplotypes but also the fact humans, with an effective population size of only 10^4^, have nearly 3 million frequent SNPs.

**Fig. 1:**
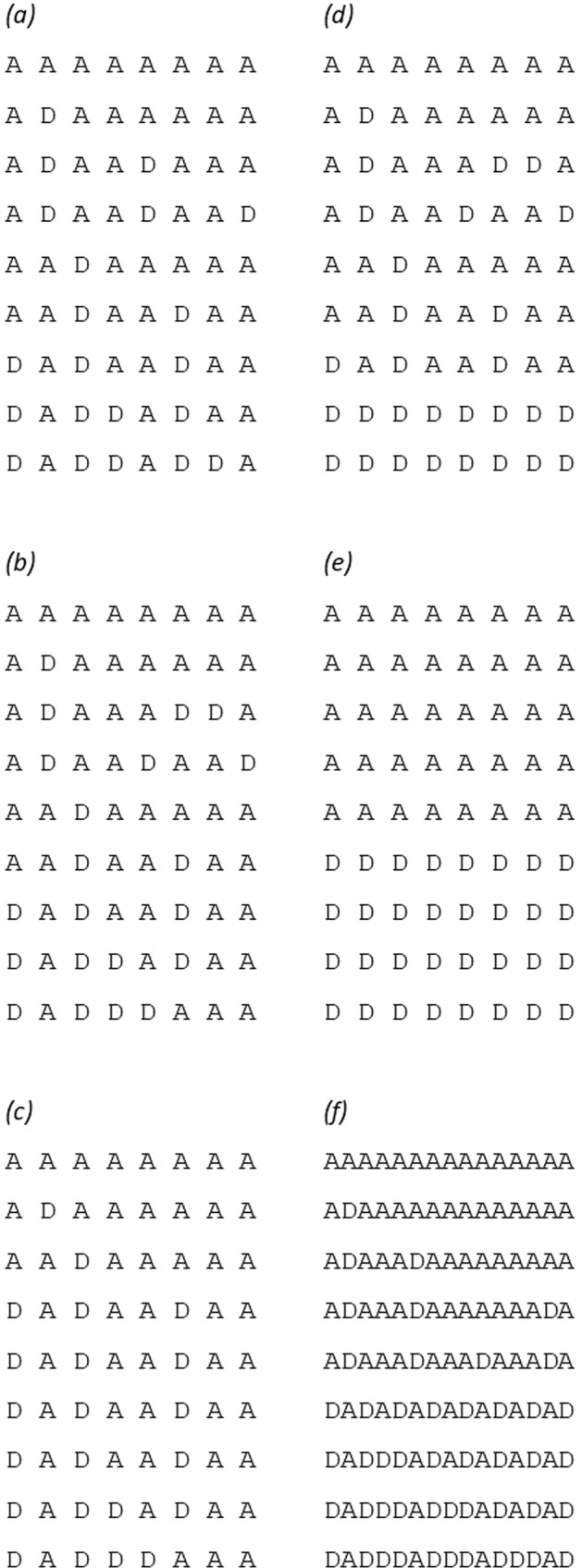
The figure portrays examples of different kinds of haplotype pattern, with each observed haplotype displayed horizontally and consisting of a mixture of ancestral (A) and derived (D) alleles. **a** Pattern expected from sequential mutational events. **b** Pattern from mutational and recombination events. **c** One haplotype increases in frequency, for example if under selection. **d** Yin yang haplotypes, pairs can be observed which differ at every locus. **e** Exclusive yin yang haplotypes, all consisting of one or the other of a yin yang pair. **f** Subsequent mutational events form new variants meaning that the variants forming the exclusive yin yang pair are no longer consecutive.

We note that in theory it is conceivable to imagine a yin yang haplotype pair such that one haplotype would consist of a mixture of reference and alternate alleles with the other haplotype having the complementary allele at each site. For common variants in humans, which allele is designated as reference and which as alternate is fairly arbitrary and the reference allele is not necessarily either the ancestral allele nor the more frequent allele but rather the allele first encountered when the initial draft of the human genome was produced. Thus if the human reference genome were based on only a single subject who was heterozygous for a yin yang haplotype pair then it would be possible that within one of the haplotypes some alleles could have been designated reference and others alternate. However if additional subjects were sequenced across this region then one would soon encounter a subject homozygous for one of the haplotypes and we might expect that all the alleles within it would then be given the same designation, whether reference or alternate. Thus, in practice it might be somewhat unusual for one member of a yin yang haplotype pair to consist of a mixture of reference and alternate alleles. Bearing this in mind and in order to accelerate and simplify our investigations, we decided to use a somewhat restricted definition of a yin yang haplotype pair as consisting of one haplotype containing only reference alleles and the other only alternate alleles.In order gain a better understanding of nature and frequency of yin yang haplotypes, we carried out a systematic search of the 1000 Genomes dataset such that all variants within each haplotype would either have the reference allele or the alternate allele but no haplotypes would carry a mixture of reference and alternate alleles. We refer to a pair of yin yang haplotypes for which intermediate haplotypes, consisting of a mixture of both, are very rarely or never seen in the population as being “exclusive”. If pair of exclusive yin yang haplotypes initially existed but then subsequent mutational events occurred then these new variants would break up the haplotypes and the variants forming the underlying yin yang haplotypes would not be consecutive. The relevant scenarios are illustrated diagrammatically in Fig. 1.

## Materials and methods

### Samples

The unphased high coverage 1000 Genomes data aligned to human reference GRCh38 was downloaded from the ftp site (http://ftp.1000genomes.ebi.ac.uk/vol1/ftp/data_collections/1000G_2504_high_coverage/working/20201028_ 3202_raw_GT_with_annot/). Subjects who were children of other subjects were excluded from the main analyses, meaning that data from 2504 subjects was used. The 1000 Genomes subjects are derived from 26 populations, which are grouped into 5 superpopulations denoted AFR, AMR, EAS, EUR and SAS.

### Pairwise genotype distances

All biallelic autosomal and X chromosome SNPs with minor allele frequency (MAF)> = 0.1 were extracted. For each of these variants, an average genotype distance was calculated between it and the next ten variants. The average genotype distance between a pair of variants was taken to be the sum of absolute differences between the number of alternate alleles carried by each subject at the two loci divided by the number of subjects. This crude measure is much quicker to calculate than formal LD statistics and although it ignores the phase relationships of doubly heterozygous subjects it is reasonable to assume that pairs of variants with average genotype distance close to zero will have LD, as measured by R^2^, close to 1. To simplify explanation of the processes followed, we will henceforth refer to the genotypes of each SNP in terms of the reference and alternate alleles as RR, RA or AA regardless of the actual nucleotides involved.

### Exclusive yin yang haplotypes

In order to find chains of variants all having identical or almost identical genotypes, these distance tables were searched beginning with each variant and continuing the chain as long a variant within the next ten could be found with an average genotype distance of no more than 0.0015. If genotypes were available for all 2504 subjects, this would imply that the genotypes of a pair of variants differed by one allele in no more than 3 subjects. Exclusive yin yang haplotypes were arbitrarily defined as chains of 20 or more of such variants having identical or almost identical genotypes, each pair being separated by no more than 9 other SNPs, and these haplotypes were retained for further investigation. The haplotypes making up these pairs would each consist of entirely reference or entirely alternate alleles. The first SNP in the haplotype is henceforth referred to as the lead SNP.

A number of chromosomal regions have been identified as having a high degree of variability and are documented as having alternate sequences [6]. A list of 545 autosomal and X chromosome known alternate sequences and patches for GRCh38 was downloaded (https://ftp.ncbi.nlm.nih.gov/genomes/all/GCA/000/001/405/GCA_000001405.29_GRCh38.p14/GCA_000001405.29_GRCh38.p14_assembly_regions.txt). Any putative yin yang haplotypes which overlapped with any of these regions was discarded and not subject to further consideration.

### Genotype counts and read depths

For each lead SNP, the number of subjects having genotype RR, RA or AA was obtained. In order to assist in identifying possible artefacts due to unrecognised repeat sequences, for each lead SNP the average number of reads called for each allele across all 1000 Genome subjects (including the children) was obtained separately for each genotype call (RR, RA or AA). We also obtained the standard deviation of the number of reads and the average allele balance (alternate allele reads divided by all reads) and the standard deviation of the allele balance.

### Recombination rates

The average recombination rate over each pair of yin yang haplotypes was assessed using the recent high resolution map based on a combination of microarray genotype and whole-genome sequence data from parent-child pairs [7]. This was also used to obtain a genetic length for the haplotypes measured in cM.

### Background variation by haplotype

The background variation associated with yin yang haplotypes was assessed in a number of ways. For each autosomal haplotype, counts were made of the number of singleton or doubleton biallelic SNPs occurring within the haplotype, with a singleton being defined as a variant observed only once across all subjects and a doubleton as a variant observed only twice. These counts were then converted to a density of variants per megabase per subject in each of the superpopulations. For each commoner SNP seen within the haplotype region (including those forming the haplotypes) the heterozygosity was calculated as 2*MAF*(1-MAF) and these were summed to obtain a “total heterozygosity” across the haplotype before being converted to an average heterozygosity per megabase in each superpopulation. These singleton and doubleton densities and average heterozygosities were also calculated from the regions of the genome not falling within a yin yang haplotype.

### Superpopulation allele frequencies and fixation index

For each lead SNP the MAF in each of the 1000 Genomes superpopulations was obtained. In addition, the global fixation index (F_ST_) was calculated according to the following formula, taking *p*_*i*_ to be the MAF for each superpopulation, *c*_*i*_ the relative sample size of each superpopulation and $\bar p$ to be the MAF for the whole sample:

$$F_{ST} = \frac{{\bar p\left( {1 - \bar p} \right) - {\sum} {c_ip_i\left( {1 - p_i} \right)} }}{{\bar p\left( {1 - \bar p} \right)}}$$

in order to allow for the possibility that some variants might differ dramatically in frequency between the African superpopulation and those outside Africa simply as a result of the out of Africa bottleneck, two versions of the F_ST_ were calculated, one using all superpopulations and one using all except the African superpopulation. For each haplotype a control SNP not in a yin yang haplotype but with the same MAF and on the same chromosome was selected and the same F_ST_ calculations were applied to these control SNPs.

### Ancestral origin of alleles

In order to investigate the ancestral origins of the alleles forming the yin yang haplotypes, we obtained autosomal variants called from whole genome sequence data for chimpanzee, Neanderthal, Denisovan and humans aligned to GrCH38 from the first author of the recent paper describing analysis of these genomes [8]. We restricted attention to variants called in both the chimpanzee and the Altai Neanderthal and to haplotypes containing at least five such informative variants. Each variant in a yin yang haplotype was placed into one of four categories depending on whether the alternate allele was observed in neither the chimpanzee nor Neanderthal, or one of them, or both. Then each haplotype was characterised according to the categories of the variants comprising it. For example, if for all variants both the chimpanzee and Neanderthal always carried the alternate allele or always carried the reference allele then this would be consistent with one haplotype being ancestral and the other derived after the human-Neanderthal split. Whereas if the chimpanzee carried all alleles of one haplotype and the Neanderthal all alleles of the other then this would suggest that the haplotypes were ancient, predating the human-Neanderthal split. If either the chimpanzee or Neanderthal carried all alleles of one haplotype but the other carried only some alleles of the other haplotype then this would imply that a partial form of the second haplotype had existed before the split and that subsequently new variants had occurred to produce the full haplotype.

### Genes and gene ontology analysis

For each haplotype we obtained the symbol of any RefSeq gene for which the region between the start of the first exon and the end of the last exon overlapped with the haplotype. We obtained the mean F_ST_ for haplotypes which did and did not overlap genes. We entered the genes which overlapped a yin yang haplotype into GOseq to carry out gene ontology analysis, incorporating the gene lengths to avoid the selection bias produced by the fact that a haplotype would by chance be more likely to overlap a long gene than a short one [9].

### Phenotypic associations

A list of variants previously reported to be associated with a variety of phenotypes in genome wide association studies (GWAS) was downloaded from the GWAS catalogue (https://www.ebi.ac.uk/gwas/docs/file-downloads, *gwas_catalog_v1.0-associations_e106_r2022-07-09.tsv*) and for the lead SNP in each yin yang haplotype a list of associated phenotypes was obtained.

### Data manipulation and statistical analyses

Data was extracted and analysed using tabix, plink and custom code written in R and C++ [10–12]. Variant identifiers to be used with the GWAS catalogue were obtained using variant effect predictor (VEP) [13].

## Results

Using the defined criteria of a minimum average genetic distance between pairs of variants of 0.0015, pairs being separated by no more than nine other variants and haplotypes needing to contain at least 20 variants, 5,779 pairs of putative yin yang haplotypes were discovered. However 461 of these overlapped with already documented GRCh38 alternate scaffolds and patches and these were discarded. Additionally, the lead SNPs for six haplotypes had genotypes called in fewer than 2000 subjects and these were also discarded.

Strikingly, there were 86 putative yin yang haplotypes which were called as heterozygous in all 2504 subjects, an obvious biological impossibility, while an additional 97 had either zero or very few calls for one or both homozygote genotypes (e.g. 7 RR, 2243 RA, 253 AA). These apparent haplotypes, all with heterozygosity above 0.53, presumably represent repeat sequences so that the bases which are called as allelic variants of each SNP are in fact variants which differ between almost identical sequences which the genotyping algorithm maps to the same position [14]. This theory is borne out by the fact that, using genotypes called as heterozygous, these lead SNPs had higher read depths (mean = 74.3, sd = 34.6) than the lead SNPs for the other haplotypes (mean = 33.9, sd = 10.8). Additionally, while the allele balance for heterozygote calls for the other lead SNPs was always close to 0.5 (mean = 0.5, sd = 0.03), the heterozygote allele balance for these putative repeats was more diverse (mean = 0.5, sd = 0.11), ranging from 0.26 to 0.84, consistent with possibility that more than two repeat sequences were present in some of these regions. Another 15 haplotypes had heterozygosity less than 0.53 but had fewer than 20 AA homozygotes and also tended to have high read depths. All these apparent repeats were not considered further, leaving a total of 5,114 exclusive yin yang haplotypes.

By the criteria used, each yin yang haplotype pair needed to consist of at least 20 SNPs. The mean number of SNPs making up each yin yang haplotype was 34.8 (sd = 19.6) with the maximum number being 233 at 4:98071252–98142231. The mean length of each yin yang haplotype was 15.7 kb (sd = 16.9 kb) with the maximum length being 268.5 kb at 3:121056668–121325164 (consisting of 199 SNPs). There were 32 haplotypes with length longer than 100 kb. In total, these exclusive yin yang haplotypes cover 80 Mb, or about 2.6% of the whole human genome.

The average recombination rate over yin yang haplotypes was 0.061 cM/Mb, some twenty-fold lower than the genome average, with the maximum being 23.62 at 19:9129001–9133768. This haplotype pair consists of 24 SNPs and has a genetic length of 0.1126 cM. It includes the interval 19:9131156–9136013, which is documented to have a recombination rate of 41.23 cM/Mb [7]. This was the highest observed genetic length and the average genetic length of yin yang haplotypes was 0.0005 cM. There was a weak, though statistically significant, negative correlation between recombination rate and physical length, r = −0.07, *p* = 4.7 × 10^−07^.

Table 1 shows the density of singleton and doubleton variants within yin yang haplotypes and in the rest of the genome. It can be seen that for the singleton variants the densities within yin yang haplotypes are similar to elsewhere in the genome. These likely reflect quite recently formed variants which have occurred at random in the time since the yin yang haplotypes formed. The number of singleton variants is expected to scale approximately linearly with the number of subjects and the fact that the densities (expressed as variants per megabase per subject) are similar across superpopulations is consistent with this. For the doubleton variants the densities are slightly lower within the haplotypes than elsewhere. There is also more variation in density between superpopulations, with the density for AFR being nearly twice as high as for EUR. This may reflect the expectation that both members of a doubleton pair will be more likely to be observed within the same superpopulation, meaning that larger superpopulations will have a higher frequency of doubletons than smaller ones. The table also shows the average heterozygosity based on all variants and shows that this is somewhat higher within the yin yang haplotypes. This may simply reflect that these regions have been selected on the basis of containing a number of common variants. This average heterozygosity, expressed as the total of heterozygosities of all variants per megabase, is expected to increase with the number of samples, but not linearly, and again this seems to be consistent with the observed differences between superpopulations.

**Table 1:** The table shows the density of singleton and doubleton variants, expressed as variants per megabase per subject, within yin yang haplotypes and across the rest of the genome in the five superpopulations.

Super-population	Number of subjects	Singleton density		Doubleton density		Average heterozygosity	
		Within yin yang haplotypes	Outside yin yang haplotypes	Within yin yang haplotypes	Outside yin yang haplotypes	Within yin yang haplotypes	Outside yin yang haplotypes
AFR	1229	8.06	8.07	3.60	4.09	1939.72	1183.53
AMR	179	7.17	7.27	2.44	2.73	1661.04	975.27
EAS	104	7.70	7.82	3.30	3.64	1458.27	904.03
EUR	503	6.16	6.05	2.00	2.25	1618.80	968.04
SAS	489	7.64	7.75	3.30	3.69	1673.01	991.38

Also shown is the average heterozygosity, defined as the total heterozygosities of all other variants per megabase, within and outside yin yang haplotypes.

The global F_ST_ mean for the lead SNPs of the haplotypes was 0.088 (sd = 0.084), which was similar that seen in the control SNPs outside yin yang haplotypes with mean 0.083 (sd = 0.079) and to that generally reported for variants in HapMap and 1000 Genomes [15, 16]. Some haplotypes exhibit remarkable differences in allele frequency between superpopulations and the maximum F_ST_ was 0.837 but similar results were seen for the control SNPs, which had a maximum F_ST_ of 0.847. The haplotypes with the highest F_ST_ values tend to have large differences in allele frequency between African and non-African superpopulations. When the African superpopulation was excluded the F_ST_ had mean 0.045 (sd = 0.045) with a maximum value of 0.426 while for control SNPs the mean was 0.044 (sd = 0.046) with maximum 0.537. There were 74 yin yang haplotypes for which the non-African F_ST_ exceeded 0.2 but these did not share any consistent patterns of allele frequency differences between superpopulations and 62 control SNPs also had non-African F_ST_ exceeding 0.2. There was a weak correlation between the non-African F_ST_ and haplotype length (R^2^ = 0.060, *p* = 2.0 × 10^–5^).

There were 4,659 autosomal haplotypes which contained at least 5 variants called in both the chimpanzee and Neanderthal. Of these, there were 76 for which the chimpanzee and Neanderthal both carried all alleles of one haplotype, suggesting that the second haplotype might have been entirely derived in humans (resembling the scenario depicted in Fig. 1). There were 16 haplotypes for which the chimpanzee carried all alleles of one haplotype and the Neanderthal all alleles of the other, implying that both haplotypes were ancient, predating the human-Neanderthal split. The possibly human-derived haplotypes were on average longer than the others, mean 39.3 kb versus 15.1 kb, *p* = 5.5 × 10^–9^. Somewhat surprisingly, the apparently ancient haplotypes were also longer than the others, mean 30.0 kb versus 15.4 kb, *p* = 0.047. In general there was no suggestion that these 16 haplotypes, one of which consisted of chimpanzee alleles and the other of Neanderthal alleles, had been produced by Neanderthal introgression because in terms of MAF in the African super-population only 3 had a value less than 0.1 and only 1 had value less than 0.01. Notably, the longest of these haplotypes consisted of 20 variants stretching over 104.3 kb at 6:70414826–70519167 with the Neanderthal carrying all reference alleles and the chimpanzee carrying all alternate alleles and this had African MAF = 0.29. It is possible that for some haplotype pairs both members might have been segregating in Neanderthals and then whether we would classify it as ancient or human-derived would depend on which haplotype happened to be observed in the single Neanderthal genome which we used.Thus, we cannot be sure that all of these haplotypes were correctly assigned. Aside from these apparently derived and apparently ancient haplotypes, the other 4567 consisted of a mixture in which partially formed haplotypes could be observed in the chimpanzee or Neanderthal or both. These partial forms provide strong evidence that yin yang haplotypes are not in general produced by a multiple mutation event in which all the constituent alleles are formed simultaneously. Moreover, 248 of these haplotype pairs contained at least one variant in which the reference allele was seen in the chimpanzee and the alternate in the Neanderthal along with at least one other variant with the reverse configuration. These pairs with apparent mixed ancestry suggest that the haplotypes we now observe in modern humans might have arisen following recombination events between earlier haplotypes.

All the above information about the haplotypes is detailed in Supplementary Table 1.

There were 2,171 (42.5%) of the haplotypes which intersected with at least one gene. This is about the same as the fraction of the genome covered by the genes used for this analysis, showing that there is no tendency for yin yang haplotypes to be either more or less common in genic regions. Interestingly, although the F_ST_ statistic is sometimes used to suggest that a locus may be under selection, there was no significant difference in the non-African F_ST_ statistic between haplotypes which did and did not overlap with genes. When the intersected genes were entered into GOseq, there was no enrichment for any gene ontology term which was statistically significant after correction for the number of terms tested.

42 of the lead SNPs were present in the GWAS catalogue and they are listed in Supplementary Table 2. They show association with a wide variety of different phenotypes but it is difficult to draw firm conclusions from these because there are at least two potential sources of bias. One is that there will be a tendency for GWASs to have identified more associated SNPs for phenotypes for which large samples have been analysed and the other is that if the lead SNP for a haplotype has an allele frequency which varies dramatically with ancestry then it may be more likely that an association will have been reported for it, possibly due to inadequately controlled population stratification. Thus it is not really possible to say with any degree of confidence whether particular phenotypes tend to be associated with yin yang haplotypes but there were no particularly obvious or striking patterns.

## Discussion

We demonstrate that exclusive yin yang haplotypes, consisting of chains of reference or alternate alleles in complete or almost complete LD with each other, are not uncommon and according to the criteria we have used to define them they cover over 2% of the human genome. We have restricted our attention to those containing at least 20 SNPs with global MAF > = 0.1 and of course if these criteria were relaxed then the number would increase. Our investigation differs from that of Dutta and colleagues in that we have focused on exclusive yin yang haplotypes where all or nearly all haplotypes are of one form or the other, whereas they studied common haplotypes for which yin yang pairs could be observed but not necessarily exclusively [3]. Other differences are that they only studied SNPs with MAF > = 0.25, that they relied on the phased haplotypes from Phase 1 of the 1000 Genomes Project and that they used data from only 1092 genomes.

For a pair of exclusive yin yang haplotypes to be observed, two conditions must be met. Firstly, the pair has to initially form, implying either that mutations at multiple sites occur simultaneously or else sequential mutations occur at different sites but subsequently intermediate haplotypes are lost, or some combination of these two processes. Secondly, after the initial formation of the haplotype pair there can be no or very few recombination events between them since these would result in novel haplotypes consisting of a mixture of reference and alternate alleles. We note that the recombination rate is indeed on average twenty-fold lower within yin yang haplotypes than elsewhere in the genome. However it is also the case that if recombination events did occur but only between reference haplotypes and/or between alternate haplotypes then the yin yang structure would still be preserved. By searching for exclusive yin yang haplotypes in which the constituent SNPs are not necessarily consecutive we have allowed for the possibility of subsequent mutational events following the initial formation of the haplotype pair. We note that singleton variants are seen at similar densities within the haplotypes as elsewhere, suggesting no marked differences in mutation rates.

We believe that the mechanism of formation of these yin yang haplotypes, which can consist of dozens of SNPs and can reach lengths over 100 kb, is not immediately obvious. The fact that most are observed in only partial forms in the chimpanzee and/or Neanderthal implies that they are not in general formed by multiple mutation events occurring simultaneously but instead must be the result of a process whereby, in the course of evolution, all intermediate forms of the haplotypes are lost.

The MAF > = 0.1 criterion we have used within 1000 Genomes means that in practice the haplotype pairs need to have a global distribution and hence predate the migration out of Africa. As previously stated, in order to remain intact since then there needs to have been no or very little recombination between members of the pair and it is unclear whether any particular molecular mechanisms contribute to this. A micro-inversion could have this effect and although we discarded regions where alternate scaffolds and patches were documented it is possible that as our knowledge of the human genome increases we may have a better understanding regarding whether recombination is specifically suppressed in a way which accounts for the preservation of some of these haplotypes.

Although individual long haplotypes may be taken as being a signal of recent selection, selection on a single haplotype is not expected to give rise to yin yang haplotype pairs. It is possible to envisage scenarios whereby selection could drive selection for a pair of haplotypes. For example, a new, advantageous allele might be formed on the background of one haplotype and subsequently a single recombination event might transfer that allele to a different haplotype or, as previously suggested, advantageous mutations might separately occur on two different haplotypes [5]. Thereafter, the two haplotypes could both increase in frequency and all alleles which differed between the original haplotypes would form a pair of exclusive yin yang haplotypes. However, in general there seems little to support the notion that the formation of exclusive yin yang haplotypes is driven by selection pressures. The distribution of the global F_ST_ for the haplotypes is not markedly different from that generally seen for other variants in 1000 Genomes and HapMap, they do not seem to preferentially impact genes and the genes which they do impact are not enriched for particular ontology terms.

An alternative explanation for the production of yin yang haplotypes which has been previously proposed is that they result from admixture events between two ancestral populations which had evolved separately over an extended period of time [1–3]. Dutta and colleagues noted the abundance of common yin yang haplotypes and “mosaic” haplotypes made up from fragments of yin yang haplotypes and postulated that their existence, along with the high level of common variation in human genomes, could be explained by a “Great Admixture” between two ancestral populations [3]. However under this model one would not expect to see exclusive yin yang haplotype pairs with one haplotype seen in chimpanzees and the other in Neanderthals nor pairs in which one haplotype appeared entirely ancestral, being seen in both chimpanzees and Neanderthals, and the other derived, being seen only in modern humans. It is also not clear that this explanation is compatible with other observations, including the geographical distribution of the haplotypes and the fact that a large number of haplotypes seem to contain a mixture of chimpanzee and Neanderthal alleles. Other scenarios leading to the production of exclusive yin yang haplotypes might involve contributions from bottlenecks, more complex effects of selection and/or additional mechanisms which are as yet unclear.

To conclude, we document the presence of exclusive yin yang haplotypes which are widespread in the human genome. It is unclear what mechanisms have led to their formation or whether they are associated with any functional significance. Their existence may challenge conventional models for how variation arises and spreads within human populations. If nothing else, because they consist of long sequences of DNA which are always inherited intact, they may be useful as markers for studies investigating the distribution of chromosomal regions through human history.

### Supplementary information

Supplementary tables

## Data Availability

Coordinates of yin yang haplotypes and their constituent SNPs are provided at https://github.com/davenomiddlenamecurtis/YinYangHaplotypes. The 1000 Genomes data and other files can be obtained from the sources documented in the text.

## Code availability

The scripts and code used to generate and process the data and produce the results described here are provided at https://github.com/davenomiddlenamecurtis/YinYangHaplotypes.
